# Pro-neurogenic effects of andrographolide on RSC96 Schwann cells *in vitro*

**DOI:** 10.3892/mmr.2020.11278

**Published:** 2020-06-26

**Authors:** Fuben Xu, Huayu Wu, Kun Zhang, Peizhen Lv, Li Zheng, Jinmin Zhao

Mol Med Rep 14: 3573-3580, 2016; DOI: 10.3892/mmr.2016.5717

Following the publication of the above article, an interested reader drew to the authors' attention that a pair of the data panels shown in Fig. 4 contained overlapping data. After having consulted their original data, the authors realized that a total of three panels featured in Figs. 4 and 6 had been erroneously selected. The image of hematoxylin and eosin (H&E) staining for RSC96 Schwann cells in the 1.5625 µM andrographolide for 6 days data panel in Fig. 4 (bottom row, second panel from the left) was mistakenly submitted. In addition, in Fig. 6, the images of immunohistochemical staining for RSC96 Schwann cells in the 3.125 µM for 4 days panel (middle row, second panel from the right) and the 6.25 µM for 6 days panel (bottom row, furthest panel on the right) were mistakenly submitted.

The corrected versions of Figs. 4 and 6 are shown opposite. These corrections were approved by all authors. The authors regret that these errors were featured in the paper, even though they did not substantially alter any of the major conclusions reported in the study. Morever, the authors apologize to the Editor of *Molecular Medicine Reports* and to the readership for any inconvenience caused.

## Figures and Tables

**Figure 4.**
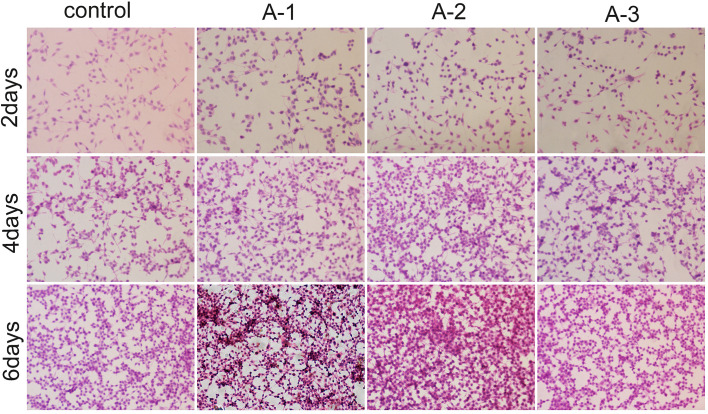
Hematoxylin-eosin staining showing the morphology of RSC96 Schwann cells cultured with 0 µM (control), 1.5625 µM (A1), 3.125 µM (A2) and 6.25 µM (A3) andrographolide for 2, 4 and 6 days. Cell seeding density: 4×10^3^/ml (original magnification, ×100).

**Figure 6.**
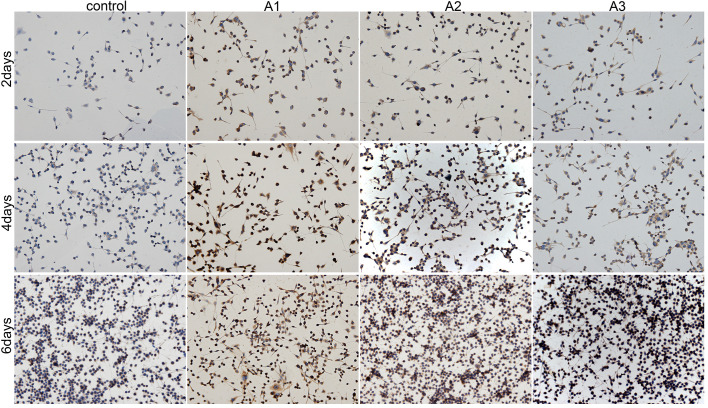
Immunohistochemical staining images showing the presence of S100β. RSC96 Schwann cells were cultured with 0 µM (control), 1.5625 µM (A1), 3.125 µM (A2) and 6.25 µM (A3) andrographolide for 2, 4 and 6 days. Cell seeding density: 4×10^3^/ml (original magnification, ×200).

